# Synthesis of nanosized nickel zinc ferrite using electric arc furnace dust and ferrous pickle liquor

**DOI:** 10.1038/s41598-021-99697-9

**Published:** 2021-10-11

**Authors:** Ayman Galal, Olfat Sadek, Moataz Soliman, Shaker Ebrahim, M. Anas

**Affiliations:** 1 Materials Science Department, Institute of Graduate Studies and Research, Alexandria University, P.O. Box 832, Alexandria, Egypt; 2 Physics Department, Faculty of Science, Alexandria University, Alexandria, Egypt; 3 EL EZZ-EL Dehkila Steel Co., Alexandria, Egypt

**Keywords:** Chemical engineering, Materials science

## Abstract

Electric arc furnace dust (EAFD) and waste pickle liquor (WPL); two major side products of the steel industry with negative environmental impact were used for the synthesis of nickel zinc ferrite (NZF); the important magnetic ceramic material of versatile industrial applications. The structural and magnetic properties of the prepared material were examined which showed good magnetic properties (high saturation magnetization and low coercivity) compared with those synthesized from pure reagents. In the applied process, nano sized nickel zinc ferrite (NZF) with a composition of Ni_x_(Zn + impurities)_1−x_Fe_2_O_4_ (where x = 0, 0.25, 0.5, 0.75 and impurities of manganese, magnesium, and calcium were prepared using zinc-containing electric arc furnace dust (EAFD) and waste pickle liquor (WPL). The chemical compositions of the prepared samples were determined using X-ray fluorescence (XRF) analysis. The optimum acetic acid concentration for EAFD treatment was found 2% v/v that decreased Ca content of EAFD by 70.6% without loss of Fe and Zn. The structural and morphological characterization was done by X-ray diffraction (XRD), Fourier transform infrared (FTIR) and Field Emission Scanning Electron Microscope (FESEM) to confirm the formation of Ni–Zn ferrite nanoparticles and estimate the particle sizes. The maximum saturation magnetization (M_s_) of 73.89 emu/g was achieved at 0.5 Ni content and the minimum coercivity of 2.55 Oe was obtained at 0.25 Ni content.

## Introduction

Zn-containing EAFD as a solid waste produced during the steelmaking process contains different valuable metals such as iron, zinc, lead, chromium, manganese, calcium, magnesium, etc. The amount of dust emitted from this process is about 15 to 20 kg per 1 ton of molten steel^1^. Because of the presence of easy leachable elements like Zn, Cd, As, Pb and other heavy metals in the EAFD its land filling is not sustainable, it represents an environmental issue. The research on EAFD are concerned with recycling of zinc from EAFD by hydrometallurgical, pyro-metallurgical and electrolysis techniques^1,2^. On the other hand, the pickling process is applied to the surface of hot rolled steel strips to remove oxide layer, making steel strips suitable for galvanizing and drawing applications. In this process, hydrochloric acid reacts with the oxide layer on the steel surface. After pickling, hydrochloric acid becomes enriched in iron in the form of ferrous and ferric chlorides and this is called “waste pickle liquor”. Waste pickle liquor is not suitable for pickling process and its discharge represents an environmental problem^3^.

Ni–Zn ferrites have a wide range of applications in the various fields of science and technology such as EMI suppressors, electromagnet cores, transformer cores, antenna, video magnetic heads and magnetic heads of multiple path communication. On the other hand, magnetic ferrite nano-particles have novel applications like therapeutic applications mainly in the drug delivery systems, in addition to catalytic applications, magnetic resonance imaging, supercapacitors and gas sensors^4–6^.

A method for preparation of Ni–Zn ferrite using EAFD was introduced by Wang et al.^7^. EAFD was treated with 0.5 mole L^−1^ HCl solution to reduce its Ca content. The treated EAFD was mixed with NiCl_2_·6H_2_O with different mass ratios with subsequent grinding and calcination at different temperatures from 800 to 1100 °C. The mass ratios of EAFD and NiCl_2_.6H_2_O along with calcination temperature influenced the magnetic properties of the prepared single-phase spinel ferrite. The maximum obtained M_s_ was 60.5 emu/g and the minimum coercivity (H_c_) was 49.8 Oe. In this work, a new preparation method for NZF was introduced using EAFD and WPL via co-precipitation technique. In this method EAFD was treated with acetic acid to reduce its Ca content, after acetic acid treatment, the EAFD was dissolved in hydrochloric acid and the leach liquor was mixed with WPL and NiCl_2_.6H_2_O followed with precipitation in NaOH solution. Co-precipitation method was selected for the proposed technique for several reasons; first is the direct usage of WPL, which is a ferric chloride liquor in the co-precipitation process. Second is making use of the dissolving step of EAFD at HCl in its purification from undesired impurities like Al and Si oxides, which have poor solubility at HCl solutions, so such impurities will not be transferred to the EAFD leach liquor that will be used for the co-precipitation process. And, finally the advantages of Co-precipitation process as a low cost method that doesn’t require high temperatures and provides crystalline nano particles with high purity in short duration.

The effect of acetic acid treatment on EAFD composition was studied and the influences of Ni ratio on the structural and magnetic properties of the prepared Ni–Zn ferrite was investigated using XRD, FTIR, FESEM and vibrating sample magnetometer (VSM) examinations. By the applied process in this paper, EAFD and WPL could be transformed from waste environmentally hazardous materials to a high added value material with both economic and environmental benefits.

## Experimental details

### EAFD treatment with acetic acid

Treatment of the EAFD with acetic acid was done to reduce the calcium oxide content. To determine the optimum acetic acid concentration for EAFD treatment, 150 mL of five different concentration acetic acid solutions (1, 2, 3, 4, 5 v/v%) were used for the treatment of five EAFD samples (15 g). The acetic acid treatment for each EAFD sample was carried at 80 °C for 1 h with a continuous stirring. The EAFD samples treated with 1, 2, 3, 4 and 5 v/v% acetic acid were denoted ATEAFD1, ATEAFD2, ATEAFD3, ATEAFD4, and ATEAFD5, respectively. The treated EAFD samples were collected by centrifugal separation at 2000 rpm and the five samples were washed with distilled water and dried at 250 °C for 4 h.

### Dissolving of the acetic acid-treated EAFD in hydrochloric acid

A 300 g sample of 2% acetic acid-treated EAFD was dissolved in 3000 mL of 15% HCl at 80 °C with a continuous stirring for 1 h. The undissolved particles were separated with centrifugal separation at 2000 rpm, and leach liquor of the dissolved sample was obtained.

### Preparation of the NZF samples by co-precipitation process

**Figure 1:**
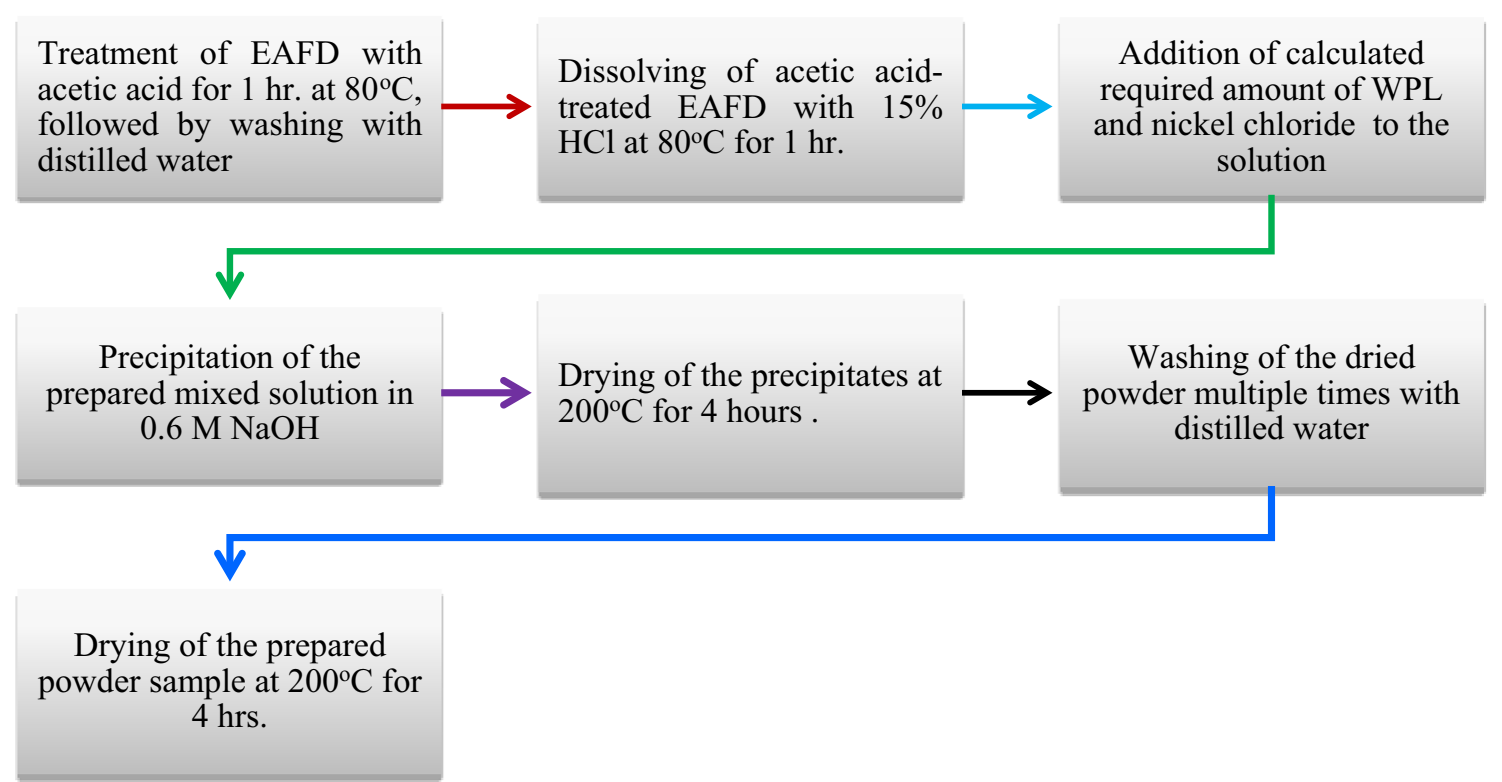
Flow chart of NZF preparation using EAFD and WPL.

Four NZF samples with the formula Ni_*x*_(Zn + impurities)_1-*x*_Fe_2_O_4_ (where *x* = 0, 0.25, 0.5 and 0.75 and the impurities contains manganese, magnesium and calcium were prepared by co-precipitation technique. First, the mixed solutions were prepared by adding NiCl_2_·6H_2_O and WPL in stoichiometric ratios to four 200 mL solutions of the leach liquor obtained from dissolving of the acetic acid-treated EAFD in HCl. Each mixed solution was added to 0.6 M NaOH solution dropwise with stirring of speed 50 rpm at room temperature. NaOH solutions were prepared taking the ratio Fe:Na to be 1:4 for each NZF composition. pH 10 was kept during the precipitation process by continuous titration with NaOH. During the addition, dark grey precipitates were obtained. The supernatant liquors were decanted, and the remaining suspensions were placed into a dryer at 200 °C for 4 h to complete ferritization reaction. The dried precipitates were washed several times with distilled water and dried at 200 °C for 4 h. The flow chart of the preparation process is shown in Fig. 1.

### Characterization

Chemical compositions of the WPL and the leach liquor obtained by dissolving of the acetic acid-treated EAFD in HCl were obtained using ICP analysis (ICP-OES Prodigy, Teledyne Leeman Labs, USA).

X-ray Fluorescence (Rigaku Supermini 200, Japan) was used to get the chemical composition of the EAFD before and after acetic acid treatment. It was also used to determine the chemical composition of the prepared nickel-zinc ferrite samples.

For phase identification, estimation of crystallite size, and other structural parameters. XRD examination was performed using X ray-diffractometer (Shimadzu-XRD 6100) using Cu K_∝_ (λ = 1.54060 Å) radiation, with continuous scan mode and scanning speed of 12 degree/minute for scanning range of 5–80 degrees. FTIR examination for the prepared nickel-zinc ferrite samples was carried out in the range of 4000–200 cm^−1^ using FTIR-8400S, Shumadzu, Japan device. The dried samples were pressed with KBr matrix, and spectra measurements were according to transmittance method. Magnetic measurements were carried out at room temperature with a maximum magnetic field of 20,000 Oe using a Lakeshore vibrating sample magnetometer (model: 8600 series VSM, lakeshore, USA) and magnetic parameters including M_s_, H_c_ and remanence (M_r_) were evaluated. NZF samples were subjected to SEM examinations using JEOL scanning microscope (JSM-IT200 Series).

## Results and discussion

### Chemical composition of WPL

The chemical analysis of WPL is shown in Table 1. It is seen that the WPL contains mainly Fe in the form of ferrous/ferric chlorides and that Mn concentration is very low so it can be used directly for the synthesis of nickel-zinc ferrite.

**Table 1:** Chemical composition of the WPL.

Constituent element	Quantity (g/L)	Error%
Fe	140	± 0.15
Mn	0.36	± 0.009

### Effect of acetic treatment on EAFD composition

**Figure 2:**
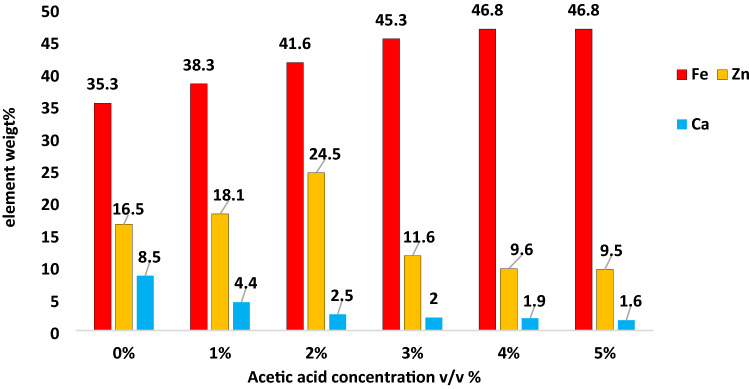
Effect of acetic acid treatment on chemical composition of EAFD.

The chemical composition of the EAFD before and after leaching with acetic acid is given in Table 2. It is seen that the effect of acetic acid treatment on Fe, Zn and Ca concentrations is most obvious than its effect on the other EAFD components. Figure 2 shows the effect of acetic acid concentration on Fe, Zn and Ca concentration in the EAFD. With increase of acetic acid concentration, iron concentration is increased while calcium concentration is decreased. For low acetic acid concentrations of 1 and 2 v/v%, Zn concentration is raised while Zn concentration is declined at higher acetic acid concentrations of 3, 4, and 5 v/v%. CaO is removed upon treatment with acetic acid due to its reaction with acetic acid according to reactions (1) and (2)^8,9^.

1 $$ {\text{CaO}} + {\text{H}}_{{2}} {\text{O}} \to {\text{Ca}}\left( {{\text{OH}}} \right)_{{2}} , $$

2 $$ {\text{Ca}}\left( {{\text{OH}}} \right)_{{2}} + {\text{ 2CH}}_{{3}} {\text{COOH}} \to {\text{Ca}}\left( {{\text{CH3COO}}} \right)_{{2}} + {\text{ 2H}}_{{2}} {\text{O}}. $$

**Table 2:** Chemical composition of the EAFD.

Constituent element	EAFD composition (wt%)	Acetic acid v/v%					Error%
		1	2	3	4	5	
Fe	35	38.3	41.6	45.3	46.8	46.8	± 0.26
Zn	16.5	18.1	24.5	11.6	9.6	9.5	± 0.35
Ca	8.5	4.4	2.5	2	1.9	1.6	± 0.40
Mg	2.8	2.1	1.7	1.7	1.7	1.7	± 0.17
Si	2.5	3	3	3.5	3.6	3.6	± 0.30
Mn	1.6	1.6	1.5	1.5	1.7	1.7	± 0.11
Pb	1.1	0.05	0.06	0.07	0.08	0.07	± 0.024
Al	0.8	0.9	1	1	1.1	1.2	± 0.06
K	0.4	0.2	0.15	0.16	0.2	0.16	± 0.0003
Cu	0.3	0.3	0.3	0.4	0.4	0.4	± 0.030
S	0.2	0.1	0.1	0.1	0.1	0	± 0.010
Ti	0.1	0.1	0.1	0.1	0.1	0.1	± 0.030
P	0.1	0.05	0.06	0.07	0.08	0.07	± 0.024

In addition, acetic acid treatment depresses Pb weight percent from 1.1% ± 0.024% in the raw EAFD sample to 0.06% ± 0.024 in the 2% acetic acid treated sample due to formation of lead acetate.

**Figure 3:**
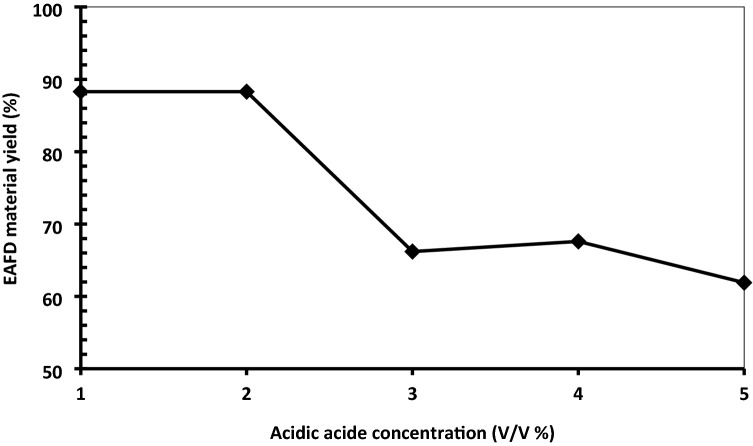
EAFD yield for the treated samples with different concentrations of acetic acid.

The EAFD material yield after the acetic acid treatment with different concentrations was calculated from the weight difference between the EAFD samples before and after treatment. As indicated from Fig. 3, the highest yield is 88.3%, which obtained at acetic acid concentrations of 1 and 2% v/v.

The best acetic acid concentration accordingly is 2 v/v% which reduced the Ca content by 70.6% ± 0.40% (from 8.5 to 2.5%) with the maximum zinc enrichment (24.5% ± 0.35) in addition to the maximum EAFD material yield (88.3%).

**Figure 4:**
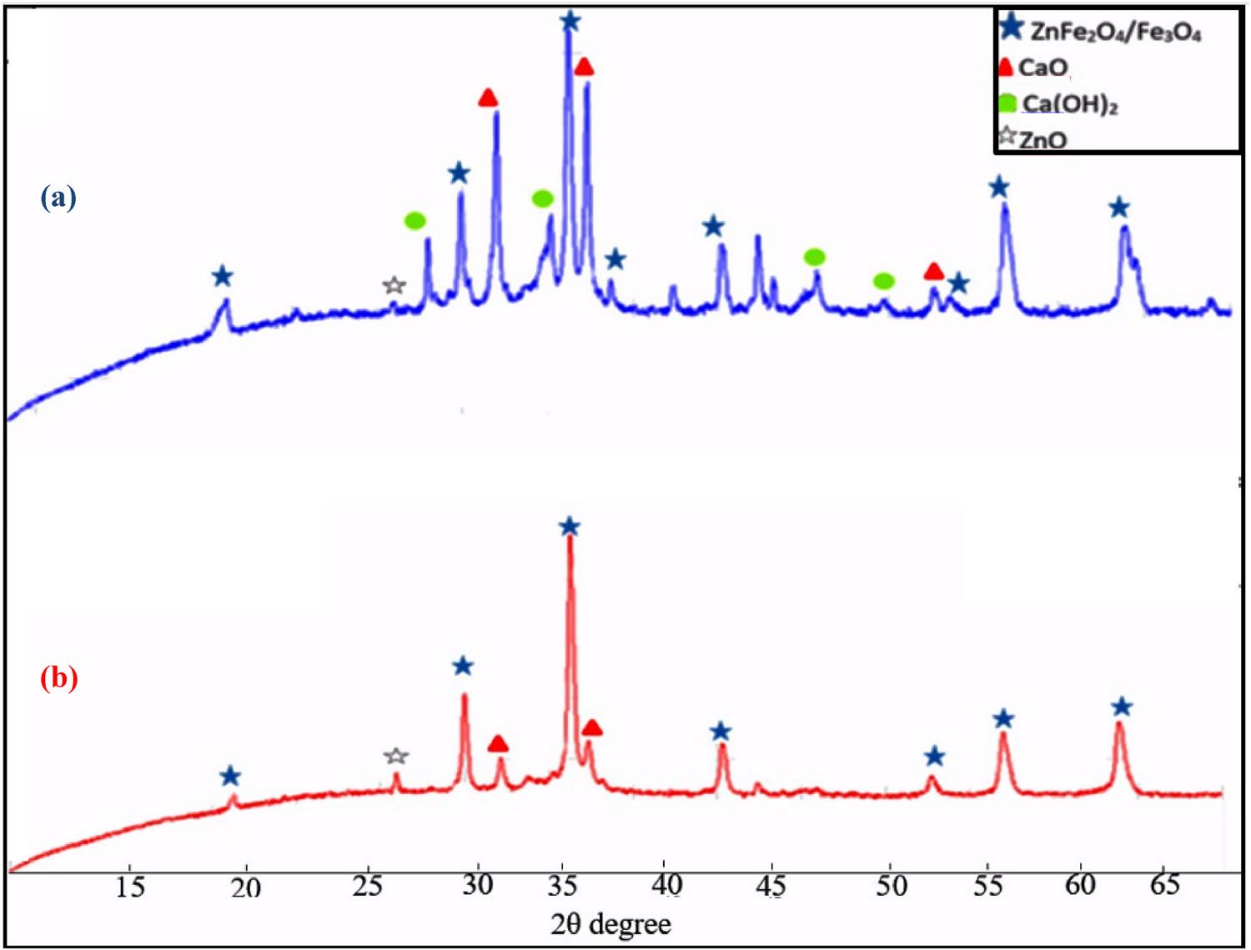
XRD patterns of EAFD (**a**) and 2% v/v acetic acid-treated EAFD (**b**).

The XRD patterns of the EAFD before and after 2% v/v acetic acid-treatment are shown in Fig. 4a,b, respectively. Untreated EAFD sample shows the diffraction peaks at 2θ around 19.2°, 29.9°, 35.1°, 36.6°, 42.7°, 53°, 56.5° and 62°. These peaks are characteristic of spinel ferrites^10^. On the other hand, the peaks of CaO are appeared at 2θ of 32.2, 36, 53.2°, and Ca(OH)_2_ peaks are displayed at around 28.5°, 34.3°, 47.8°, and 50.7°^11^. The XRD pattern of the acetic-acid treated sample shows that a disappearance of the Ca(OH)_2_ peaks and decrease of CaO peaks intensities are observed.

### Chemical composition of the dissolved acetic acid-treated EAFD leach liquor

The chemical analysis of the obtained liquor after dissolving of the acetic acid-treated EAFD sample in 15% HCl solution is illustrated in Table 3. It is noticed that Si and Al didn’t exist which indicates that their oxides have poor solubility in HCl. The weight of the undissolved particles is 29.23 g representing 9.74% of the acetic acid-treated EAFD sample weight. Incomplete dissolving of the sample could be due to the impurities of metal oxides with a lower solubility in HCl solution mainly silicon and aluminum.

**Table 3:** Chemical composition of the acetic acid-treated EAFD leach liquor.

Constituent element	Acetic acid treated EAFD HCl leach liquor	Error%
Fe	30	± 0.15
Zn	9.5	± 0.08
Mn	0.99	± 0.009
Mg	0.8	± 0.022
Cu	0.022	± 0.005
Ca	0.45	± 0.20
Pb	0.083	± 0.006

### Chemical analysis of the prepared NZF precipitates

The chemical analysis of the prepared samples is shown in Table 4. Although Cu and Pb are detected in dissolved acetic acid treaded EAFD solution, they are disappeared in the prepared NZF samples due to their very low concentration in the precipitation solution.

**Table 4:** Chemical analysis of the prepared NZF samples.

Element	Sample				Error%
	N_0.0_ZF	N_0.25_ZF	N_0.50_ZF	N_0.75_ZF	
%Fe	49.0	48.1	48.2	48.3	± 0.26
%Zn	17.01	13.0	8.03	3.9	± 0.35
%Mg	2.502	1.53	1.0	0.51	± 0.17
%Mn	2.53	1.51	1.0	0.42	± 0.11
%Ca	0.71	0.51	0.31	0.0	± 0.40
%Ni	0.0	6.52	12.9	19.04	± 0.25

### Structural properties of the prepared NZF samples

**Figure 5:**
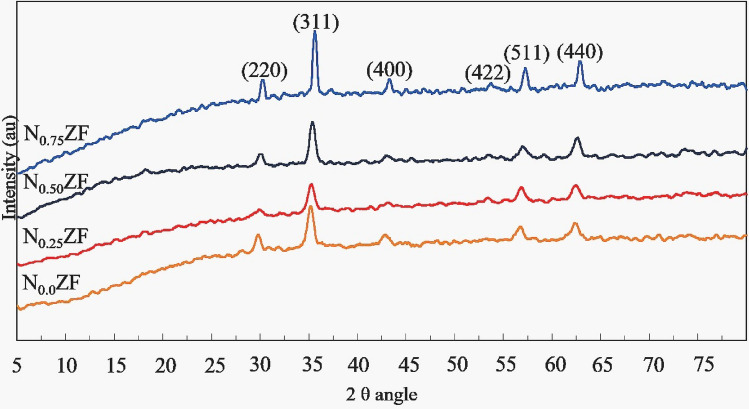
XRD patterns for the prepared NZF samples with different Ni contents.

XRD patterns of the as prepared NZF samples with different Ni contents are shown in Figure 5.

The results indicate that the reflections in all the cases are characteristic of the spinel structure without any impurity phases. There is a slight shifting of the 2θ of the diffraction peaks to higher values with increase of Ni ratio. This shifting is attributed to the fact that the size of Ni^2+^ cation is smaller than that of Zn^2+^ cation (69 Å and 74 Å respectively), so the lattice parameter of the spinel unit cell decreases with increasing of Ni content. This results in a larger diffraction angle because the diffraction angle θ is inversely proportional to the lattice parameter. The decrease of lattice constant with increase of Ni content was reported by many previous studies about the effect of Ni substitution ratio on the structural properties of NZF^12–14^.

The crystallites size (D) of NZF samples was calculated using Scherer formula^15^.

3 $$D\left(hkl\right)=\frac{0.90\uplambda }{{\upbeta \cos}\uptheta },$$

where β is the peaks full width at half maximum in radians, and λ is the wavelength of the used X-ray beam and 0.90 is the shape factor.

Peak of (311) reflection is used for calculations because it is the sharpest one. The crystallites size are 10.8, 10.7, 12.0 and 16.7 nm for samples N_0.0_ZF, N_0.25_ZF, N_0.50_ZF and N_0.75_ZF, respectively. The crystallite size is almost the same at Ni = 0.0 and 0.25, while further increase of Ni to 0.5 and 0.75 showed an obvious increase in the crystallite size. The observed increase of the crystallite size with increase of Ni content was observed by Chuan et al.^16^ and Upadhyay et al.^17^. It is attributed to Zn-ferrite formation is more exothermic than Ni-ferrite formation, so the surface temperature of the formed crystals becomes higher with increase of the Zn content which reduces the molecules concentration at the surface of the growing crystals.

The lattice parameters for each sample was calculated using the formula^18,19^.

4 $$a=d\sqrt{{h}^{2}+{k}^{2}+{l}^{2}},$$

where a is lattice parameter, d is the inter planer spacing and h, k, l are the miller indices of (311).

The lattice parameter shows a slight decrease in its value with increase of Ni content which is attributed to its lower cation size compared to Zn cation.

The theoretical XRD density ρ_x_ was calculated using the formula^20,21^.

5 $${\rho }_{x} =\frac{8M}{{A}_{N}V},$$

where M is NZF samples molecular weight, A_N_ is Avogadro’s number (mol^−1^) and V is the lattice volume. A slight increase of ρ_x_ is observed with increase of Ni ratio due to the accompanied reduced lattice parameter.

**Figure 6:**
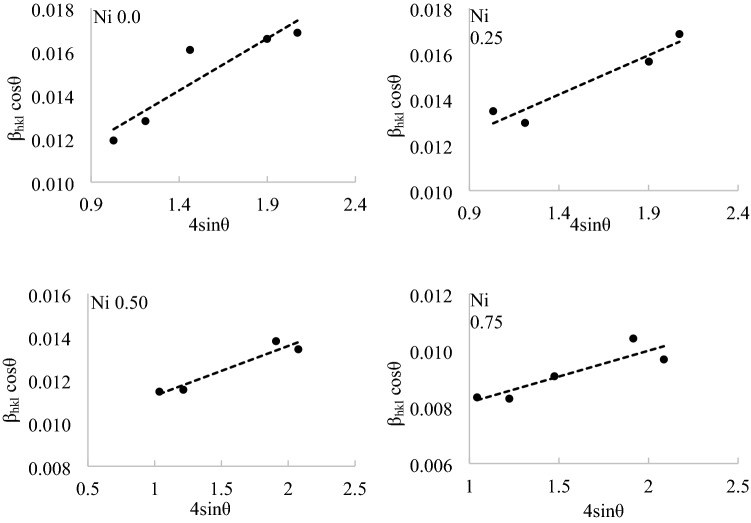
Plots of β_hkl_ cosθ vs 4sinθ of NZF samples.

The lattice strain ε was calculated using Williamson–Hall equation^22,23^.

6 $${{\upbeta_{\text{hkl}}} \cos \theta }= \frac{k\lambda }{D}+4 \varepsilon \sin \theta ,$$

the lattice strain can be estimated with the aid of Eq. (6) from the slope of β_hkl_ cosθ versus $4 sin\theta$ plot as in Fig. 6. The estimated lattice stain values declines with the increase of Ni content due to the smaller Ni^2+^ cation size which makes less distortion for the spinel lattice than the larger Zn^2+^ cations, a similar phenomenon was reported by Abu El-Fadl et al.^13^ in the study of NZF prepared by microwave combustion method and by Srinivas et al.^24^ in co-precipitated NZF and by Batoo et al. in nanoparticles of Ni–Cu–Mg ferrite prepared by sol–gel method^25^. The XRD calculated crystallites size, lattice parameter, lattice strain and XRD density for all samples are listed in Table 5.

**Table 5:** Results of the XRD test for the NZF samples.

Sample	2θ degree (311)	Lattice parameter a, Å	Crystallite size, nm	Lattice strain ε	XRD density ρ_x_, g/cm^3^
N_0.0_ZF	35.17	8.460	10.8	0.0048	4.96
N_0.25_ZF	35.23	8.422	10.7	0.0034	5.06
N_0.50_ZF	35.355	8.417	12.0	0.0023	5.06
N_0.75_ZF	35.59	8.356	16.7	0.0018	5.20

**Figure 7:**
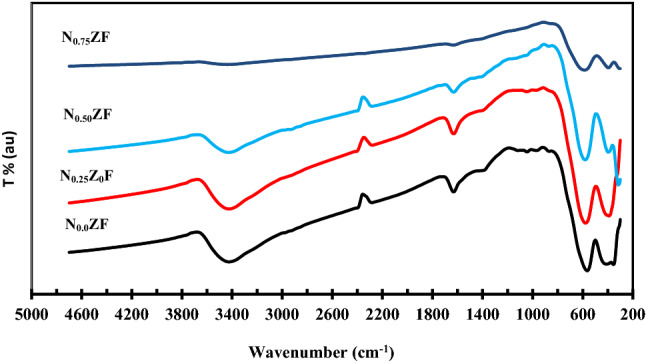
FTIR spectra for the prepared NZF samples.

FTIR spectra of the prepared NZF samples are shown in Fig. 7. Two main broad absorption bands are appeared in the range of 390 to 586 cm^−1^. The highest wavenumber one is designated ʋ1, observed in the wavenumber range of 565 to 586 cm^−1^ and this band corresponds to the metal ions intrinsic stretching vibrations at the tetrahedral site. The other band appeared in the range of 392 to 409 cm^−1^ and it is designated as ʋ2 and corresponds to metal ions stretching vibrations in the octahedral sites. These two bands are observed in previous studies about spinel ferrites^24–28^.

It is seen from Table 6 that there is a slight shifting of ʋ1 towards higher wavenumbers with increase of Ni content. This can be explained the in view of the cation distribution within NZF spinel structure. Zn ferrite has a normal spinel structure where Zn^2+^ cations occupy the tetrahedral sites (A sites) and Fe^3+^ cations occupy the octahedral sites (B sites). On the other hand, Ni ferrites have inverse spinel structure in which octahedral sites are occupied by both Ni^2+^ and Fe^3+^ cations. Accordingly, when Ni^2+^ cations are added in expense of Zn^2+^ cations, they occupy the octahedral sites pushing part of Fe^3+^ cations from the octahedral to the tetrahedral sites. As Fe^3+^ cations are smaller in size and lighter than Zn^2+^ cations, atomic vibration of the tetrahedral sites increases as well giving rise to shifting of the absorption band towards higher wavenumbers. The same shifting of ʋ1 towards higher wavenumbers with increase of Ni was also reported by Tahrani et al.^12^ and Batoo et al.^29^. Other bands are observed around 1630 and 3420 cm^−1^ and the first is attributed to deformation vibrations of adsorbed water during preparation while the second is attributed to O–H stretching indicating the remaining of some hydroxyl groups^12,30^. The intensity of these two bands are decreased with increase in heat treatment temperature.

**Table 6:** The effect Ni ratio on the wavenumber of ʋ1 and ʋ2 bands.

Sample	Wavenumber	
	υ1, cm^−1^	υ2, cm^−1^
N_0.0_ZF	565	409
N_0.25_ZF	579	392
N_0.50_ZF	580	397
N_0.75_ZF	586	397

**Figure 8:**
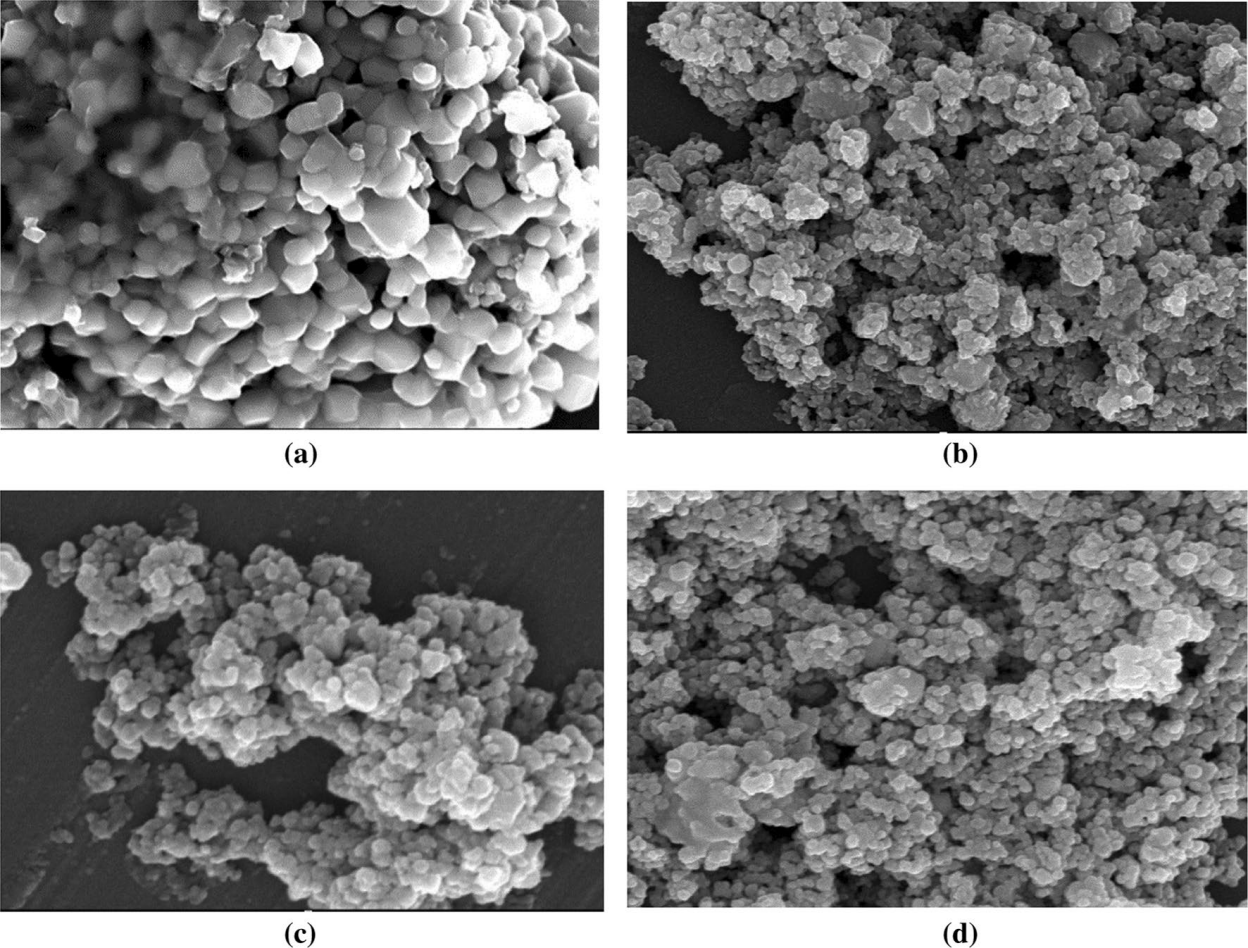
SEM micrographs of NZF samples (**a**) NZF, (**b**) N_0.25_ZF, (**c**) N_0.5_ZF and (**d**) N_0.75_ZF.

Figure 8 (from a to d) display the SEM images of the prepared NZF samples. The sample with zero Ni content has non-uniform hexagonal and spherical particles. As Ni content increase, the particles become more spherical in shape and finer in size. A similar effect of Ni content on NZF morphology was reported by Khan et al.^31^ in the study of the effect of Zn^2+^dopping on Nickel ferrites.

### Magnetic characterization

**Figure 9:**
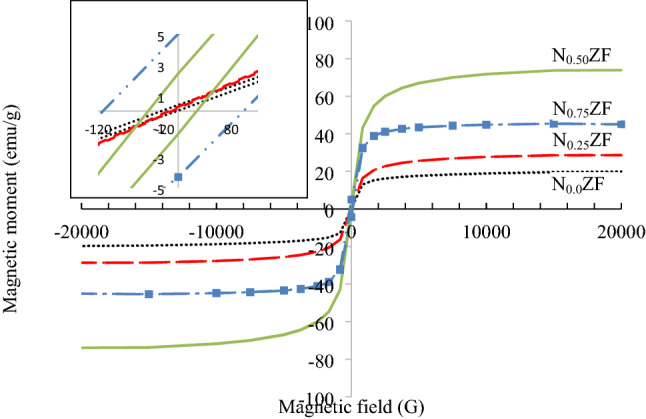
Magnetic hysteresis loops for the prepared NZF samples.

Figure 9 depicts the magnetization hysteresis loops obtained by VSM measurement for the prepared NZF samples. The obtained values of M_s_, H_c_ and retentivity (M_r_) are shown in Table 7. It is seen from Table 7 that the specific Ms values of 19.8, 28.64, 73.89 and 45 emu/g are observed for the NZF samples N_0.0_ZF, N_0.25_ZF, N_0.50_ZF and N_0.75_ZF, respectively. The saturation magnetization of magnetic ferrites depends on their chemical composition and the cations distribution within their spinel structure. In NZF spinel lattice, Ni^2+^ cations prefer to occupy the octahedral sites whereas, Zn^2+^ cations prefer the tetrahedral sites, and Fe^3+^ cations are distributed between both octahedral and tetrahedral sites^32^. The net magnetic moment of the spinel lattice is the difference between the magnetic moments of the octahedral B and the tetrahedral A sub-lattices (M = M_B_ − M_A_) where M_B_ is the magnetic moment of B sub-lattice and M_A_ is the magnetic moment of A sub-lattice^32–35^. As Ni^2+^ content at the octahedral sites increases, more Fe^3+^ cations are forced to move from octahedral to tetrahedral sites. As the magnetic moments of Fe^3+^ cations are higher than that of Ni^2+^ cations (5 μ_B_ and 2 μ_B,_ respectively_)_, the replacement of Ni^2+^ cations for Fe^3+^ cations at octahedral sites results in a decrease of M_B_ and an increase of M_A_. The increase of M_A_ with Ni addition produces a higher and super-exchange interaction between A and B sub-lattices which leads to an increase of the magnetization value. However, at a higher content of Ni up to 0.75 the reduction of net magnetic moments between A and B sub-lattices is observed and this can be elucidated based on the high super exchange interaction between the two cations sites.

**Table 7:** Magnetic parameters of NZF samples with different ratios of Ni.

Sample	M_s_, emu/g	M_r_, emu/g	H_c_, Oe
N_0.0_ZF	19.819	0.18	11.41
N_0.25_ZF	28.64	0.051	2.55
N_0.50_ZF	73.89	1.97	36
N_0.75_ZF	45	4.66	105

It is also noticed from the magnetization examinations that sample N_0.0_ZF with zero Ni content achieves 19.8 emu/g of Ms, which is abnormal as zinc ferrite should be paramagnetic at room temperature because all Fe^3+^ cations are located in the octahedral sites and all Zn^2+^ cations are localized in the tetrahedral sites. Therefore, the magnetic spins of the octahedral Fe^3+^ cations can’t be co-aligned because of the absence of super-exchange between octahedral and tetrahedral sub-lattices^36^.

The existence of Mn, Mg and Ca impurities in the prepared NZF samples can explain the saturation magnetization of the zero Ni sample where the presence of Ni modifies the cations distribution and the magnetic moments in the NZF spinel lattice. Mn^2+^ and Ca^2+^ cations prefer the tetrahedral sites in the spinel lattice, whereas Mg^2+^ cations prefer the octahedral sites^37–40^. As Mn^2+^ cations with high magnetic moment (5 μ_B_) occupy the tetrahedral sites and Mg^2+^ cations with zero magnetic moment occupy octahedral sites, M_A_ increases and M_B_ decreases. The increase of the magnetic moments of the tetrahedral A sub-lattices increases their capability to align antiparallel to the magnetic moments of the octahedral B sub-lattices and accordingly enhances the super-exchange interaction between A and B sub-lattices and increases the net magnetic moment. The abnormal saturation magnetization of the sample N_0.0_ZF can also be attributed to the cations miss distribution in nanosized ferrites. In nanosized NZF some Zn^2+^ cations may occupy the octahedral B sites instead of their most favorable tetrahedral A sites, leading to shifting of some Fe^3+^ cations to the tetrahedral sites. This abnormal cation distribution illustrates the increase of saturation magnetization in the zinc ferrite sample^13^.

On the other hand, the minimum coercivity of 2.55 Oe is observed at Ni content of 0.25 for sample N_0.25_ZF whereas sample N_0.75_ZF exhibits the maximum coercivity of 105 Oe. The high value of the coercivity at Ni content was also obtained by Abu El-Fadl et al in NZF prepared by microwave combustion method, and by Chaudhari et al in NZF synthesized by oxalate precursor method. The phenomenon of coercivity increase with increase of Ni content is attributed to the high magnetocrystalline anisotropy energy of Ni ions in comparison to Zn ions^13,40^. It is also observed from Table 7 that sample N_0.0_ZF of zero Ni content exhibits higher coercivity compared to N_0.25_ZF sample (11.41 and 2.55, respectively). It was also observed by Abu El-Fadl et al in the previous mentioned study that the sample with 0.2 Ni content exhibited lower coercivity than that with zero Ni content. This was attributed to the fact that Fe^3+^ content on the A-site is the highest for Ni content of 0.2 in comparison with Ni content of 0.0^13^.

## Conclusion

Single phase nanosized NZF was produced using EAFD and WPL. Treatment of the EAFD with 2% v/v acetic acid reduced its Ca content by about 70% and enriched its content of Fe and Zn. Dissolving of the acetic acid-treated EAFD in hydrochloric acid resulted in removal of non-soluble SiO_2_ and Al_2_O_3,_ which improved the purity of the prepared samples. The structural and magnetic properties of the prepared NZF samples are affected by their Ni content and the highest saturation magnetization of 73.89 emu/g was achieved at 0.5 Ni ratio and the lowest coercivity of 2.55 Oe was achieved for Ni content 0.25. The presence of Ca, Mn, and Mg impurities affected the magnetic properties of the prepared samples by readjustment of the cation’s distribution and the magnetic moments within the spinel NZF lattice. The applied process in this paper realized the transformation of the EAFD and WPL form environmentally hazardous waste by products to value-added magnetic ferrites.
